# Interpretable Bayesian Modeling for Multireader Multicase Studies: Addressing Overdispersion and Limited Sample Size in Diagnostic Enhancement Evaluation

**DOI:** 10.1002/sim.70666

**Published:** 2026-07-03

**Authors:** Paul‐Philipp Jacobs, Ingo G. Steffen, Constantin Ehrengut, Susann Bräuer, Hans‐Jonas Meyer, Timm Denecke

**Affiliations:** ^1^ Department of Interventional and Diagnostic Radiology University of Leipzig Leipzig Saxony Germany; ^2^ Department of Diagnostic and Interventional Radiology and Nuclear Medicine Charité–University Medicine Berlin Germany; ^3^ Department of Diagnostic and Interventional Radiology and Nuclear Medicine University Medical Center Hamburg‐Eppendorf Hamburg Germany

**Keywords:** balanced accuracy, chest X‐ray, hierarchical Bayesian modeling, multireader multicase, overdispersion

## Abstract

The advent of machine and deep learning in the medical domain has led to significant advancements in diagnostic workflows and clinical decision‐making, making rigorous evaluation of novel techniques essential for their integration into clinical practice. In this work, we introduce a Bayesian hierarchical Beta‐Binomial modeling framework for estimating the effect of novel techniques in binary classification, with a particular focus on multireader, multicase study designs, motivated by applications in medical imaging. Some challenges in this context include, small sample sizes (i.e., only few readers particicipating in the study), pronounced overdispersion due to heterogeneity in reader performance, and class‐imbalanced datasets. Addiotionally, the actual effect size of the novel technique may be small, further complicating robust estimation of model parameters. The proposed model explicitly accounts for overdispersion, addresses class imbalance within the test cohort, and incorporates prior information to regularize population‐level parameter estimates across readers. Through simulation studies, the approach demonstrates improved robustness and lower estimation error compared to classical linear models, especially under high overdispersion and low sample sizes. Application to a real‐world study of chest X‐ray imaging with and without Bone Suppression Imaging enhancement illustrates the model's practical utility and highlights the importance of accounting for overdispersion and prior information in study design and analysis.

**Abbreviations**
AUC: area under the curve
CXR: chest X‐ray
fpr: false positive rate
MRMC: multireader, multicase
ROC: receiver operating characteristic
tnr: true negative rate
tpr: true positive rate

## 1 Introduction

Recent advances in Machine Learning and Artificial Intelligence have created substantial opportunities for augmenting clinical decision‐making and diagnostic workflows through Computer‐Aided Diagnostic (CAD) systems. In medical imaging, the emergence of Convolutional Neural Networks has markedly improved performance in computer vision tasks such as semantic segmentation, object detection and image classification. These methods have been applied across a wide range of imaging modalities, including chest X‐ray (CXR) [1, 2], computed tomography [3, 4], magnetic resonance imaging [5], positron emission tomography [6, 7], and ultrasound imaging [8]. Such approaches have demonstrated potential for improving diagnostic accuracy, reducing interpretation time and enhancing patient outcomes—benefits that are particularly relevant in the context of pandemic scenarios. Consequently, rigorous and comprehensive evaluation of these techniques is essential to ensure their clinical utility and effectiveness.

The clinical adoption of novel techniques designed to augment human diagnostic performance is contingent upon robust evidence of their impact on diagnostic accuracy. Such evaluation is commonly performed in research studies that systematically compare relevant performance metrics in the presence and absence of the technical enhancement. In this work, we focus on a common and methodologically rigorous scenario: the fully crossed multireader, multicase (MRMC) study design for image‐based binary classification tasks. This design involves multiple human readers independently assessing the likelihood of a given condition across a set of cases, under both experimental conditions (i.e., standard imaging and enhanced imaging). The precise experimental setup may vary between studies, particularly with respect to the number of readers, the number of cases, and the inclusion or omission of a washout period between readings [9]. Although this study focuses on the medical imaging domain, the proposed method is applicable to any binary classification task in which the effect of an intervention or modification—such as a change in diagnostic protocol or technology—is to be estimated using a MRMC study design.

A review of MRMC studies indicates that, in the majority of cases, the number of participating readers rarely exceeds ten, except in large‐scale multicenter investigations [10]. This limitation results in small datasets and necessitates averaging over a limited sample of the population of similar or comparable (based on the sample selection criterion), which in turn leads to population parameter estimates that are inherently noisy and sensitive to extreme values. As a consequence, population‐level estimates and predictions of out‐of‐sample performance are characterized by low statistical power and substantial uncertainty.

An additional important factor is the heterogeneity in performance among individual readers, which may arise from differences in experience, expertise, or interpretation of imaging findings. Such heterogeneity introduces overdispersion into the data, thereby increasing the risk of bias in the estimation of model parameters. Although overdispersion is a well‐recognized phenomenon in MRMC studies and count data in general [11, 12], it is often challenging to mitigate this variability by restricting the reader cohort to a homogeneous group, as practical constraints may preclude the selection of readers with comparable backgrounds. Therefore, quantifying the degree of overdispersion present in the data is essential for the appropriate interpretation of statistical analyses.

Statistical assessment of classification performance in this context is most commonly conducted using Receiver Operating Characteristic (ROC) curve analysis, which quantifies diagnostic accuracy in terms of sensitivity and specificity [13]. Nevertheless, for binary classification tasks, accuracy remains a widely used and interpretable metric, particularly in studies involving human readers, as it directly reflects the proportion of correctly classified cases. Unlike ROC curve analysis, which characterizes reader performance across the entire range of possible thresholds, accuracy is evaluated at a fixed decision threshold and thus offers a more direct interpretation in clinical practice. A key advantage of the proposed framework is its ability to perform threshold‐dependent inference across the entire range of possible decision thresholds, enabling comprehensive performance evaluation beyond fixed‐point estimates. In scenarios with class‐imbalanced datasets, where the prevalence of positive and negative cases differs, balanced accuracy provides a robust alternative by correcting for bias introduced by class imbalance.

When confronted with small sample sizes, small effect sizes and pronounced overdispersion, Bayesian modeling offers a principled approach for estimating critical parameters such as balanced accuracy by explicitly integrating prior knowledge about plausible parameter value ranges and their expected variability. Employing informative prior distributions enables regularization of parameter estimates, thereby reducing the influence of sample noise and preventing implausible inferences. Such priors may be informed by previous empirical studies, expert elicitation, or domain‐specific knowledge and are particularly valuable for constraining parameters, such as the effect size of imaging enhancement techniques, for which a substantial body of comparable prior research is available. The Bayesian framework further provides full posterior distributions for all parameters, supporting probabilistic interpretation and rigorous quantification of uncertainty.

The structure of this paper is as follows: First, we present a Bayesian Beta‐Binomial model for the estimation of balanced accuracy in binary classification tasks aggregated across readers. Unlike other models the Beta‐Binomial approach does not explicitly model reader‐case interactions or case‐specific effects. These sources of variation are absorbed into the overdispersion parameter instead. Subsequently, we evaluate the Beta‐Binomialmodel's performance in a simulation study, benchmarking it against a simplified classical linear modeling approach.

To accommodate case‐level reading data, we propose a second Bayesian hierarchical model based on Bernoulli likelihood for invividual case‐level outcomes, which explicitly models case‐specific effects and reader‐case interactions. We demonstrate the application of the Bayesian model to a real‐world dataset, specifically an MRMC study assessing the diagnostic performance of pulmonary infiltrations in CXR imaging with and without Bone Suppression Imaging (BSI) enhancement. To set the results in the context of existing methods, we compare the results of the Bayesian model to those obtained using the well‐known Obuchowski‐Rockette (OR) method for MRMC studies [13].

To ensure reproducibility and transparency in the communication of our study results, we adhered to the Bayesian Analysis Reporting Guidelines as proposed by Kruschke et al. [14] wherever applicable.

## 2 Methodology

Models and methods presented in this Paper are available in the GitHub repository https://github.com/PeePeeJay/BayBacMRMC.

### 2.1 Balanced Accuracy

In binary classification tasks, *accuracy* quantifies the proportion of correctly classified instances among all evaluated cases [15]. It is defined as

(1) $$\text{Accuracy}=\frac{tp+tn}{tp+tn+fp+fn},$$

where tp and fp denote the number of true and false positive predictions and tn and fn represent the number of true and false negative predictions, respectively. However, this metric can be substantially biased in the presence of class imbalance, as classifiers may achieve high accuracy by favoring the majority class. To address this limitation, *balanced accuracy* was introduced as a metric that accounts for class imbalance by averaging the classification performance across both classes [16, 17]. Specifically, balanced accuracy is computed as the arithmetic mean of the true positive rate (sensitivity) and the true negative rate (specificity):

(2) $$\text{Balanced Accuracy}=\frac{1}{2}\left(\frac{tp}{p}+\frac{tn}{n}\right),$$

where p and n denote the total number of positive and negative cases, respectively. For a classifier that does not exploit class imbalance, balanced accuracy reduces to conventional accuracy. Conversely, if a classifier leverages class imbalance, balanced accuracy appropriately reflects this by converging to the level expected by random chance.

### 2.2 Beta‐Binomial Model for Aggregated Reading Data

For clarity, we first define the model for unbalanced accuracy before extending it to balanced accuracy. The number of correctly classified cases, k, can be conceptualized as the outcome of multiple independent Bernoulli trials. Given I readers in the study, each reader i (i=1,…,I) is characterized by a probability $\phi_{i}$ of correctly classifying a case. Accordingly, $k_{i}$ is modeled as an independent binomial random variable:

(3) ki∼Binomial(ni,ϕi)=nikiϕiki(1−ϕi)ni−ki,

where $n_{i}$ denotes the number of trials for reader i, and $\phi_{i}$ is assumed to be a random variable drawn from a Beta distribution:

(4) $$\phi_{i}∼\text{Beta}(\alpha ,\beta )=\frac{1}{B(\alpha ,\beta )}\phi_{i}^{\alpha -1}{\left(1-\phi_{i}\right)}^{\beta -1}.$$

The parameter $\phi_{i}$ represents the accuracy of reader i in the binary classification task. Marginalizing over $\phi_{i}$ yields the Beta‐Binomial distribution:

(5) $$\begin{array}{ll} k_{i} & ∼\text{BetaBinomial}(n_{i},\alpha ,\beta ) \end{array}$$

(6) =nikiBeta(ki+α,ni−ki+β)Beta(α,β).

The Beta‐Binomial distribution can be reparameterized in terms of the mean $\mu =\frac{\alpha }{\alpha +\beta }$, with 0<μ<1 and the overdispersion parameter $\gamma =\frac{1}{\alpha +\beta +1}$, with 0<γ<1. This reparameterization facilitates direct modeling of the degree of overdispersion relative to the mean in the data.

Building upon previously proposed models for balanced accuracy [16, 17], we extend the framework by explicitly incorporating the effect of an (imaging) enhancement technique. Specifically, we model $\phi_{i}$ using a logit link function:

(7) $$\phi_{i}=\frac{1}{1+e^{-(a+b\cdot x_{i})}},$$

where $x_{i}$ is a binary indicator denoting the application of the enhancement technique, a is the intercept, and b is the slope parameter. The intercept, slope, and overdispersion parameters are treated as population‐level quantities, assumed to be shared across all readers sampled from the underlying population. In this context, the intercept corresponds to the baseline population accuracy, the slope quantifies the change in accuracy attributable to the application of the enhancement technique, and the overdispersion parameter captures inter‐reader variability.

The overdispersion parameter γ controls the Beta concentration through $\alpha_{i}+\beta_{i}=(1-\gamma )/\gamma$, so that $E[k_{i}]=np_{i}$ but

(8) $$\mathrm{Var}(k_{i})=np_{i}(1-phi_{i})\left[1+(n-1)\gamma \right].$$

Thus, γ is an intraclass‐correlation‐style parameter for within‐cell dependence: γ→0 recovers the Binomial model, whereas larger γ increases variance inflation beyond Binomial sampling noise. In practical terms, γ absorbs residual heterogeneity (e.g., unmodeled case difficulty or reader‐case idiosyncrasy). This yields more conservative posterior uncertainty for treatment effects and derived performance metrics (true positive rate (tpr), true negative rate (tnr), ROC summaries) while preserving the same mean structure. This behavior is particularly advantageous when little is known a priori about the degree of reader‐case interaction or case‐level variability, as it allows the data to determine the extent of overdispersion without imposing restrictive assumptions.

This hierarchical modeling approach enables the estimation of population‐level parameters by pooling information across all readers, while still accommodating individual‐level heterogeneity. Posterior samples of a, b, and γ yield point estimates for the population mean accuracy Φ, the mean effect size η associated with the imaging enhancement and the mean overdispersion Γ as follows:

(9) $$\begin{array}{ll} \Phi & =\frac{1}{1+e^{-â}}\;, \end{array}$$

(10) $$\begin{array}{ll} \eta & =\frac{1}{1+e^{-(â+\hat{b})}}-\Phi , \end{array}$$

(11) $$\begin{array}{ll} \Gamma & =\hat{\gamma },\; \end{array}$$

where â, $\hat{b}$, and $\hat{\gamma }$ denote the posterior mode of the intercept, slope, and overdispersion parameters, respectively.

To generalize the model for balanced accuracy, the framework is applied independently to the positive and negative cases. The posterior distributions of the parameters a and b for balanced accuracy are then obtained by averaging the corresponding parameters from the positive and negative models:

(12) $$\begin{array}{cc} p(a|k_{i:I}^{+},k_{i:I}^{-}) & =p\left(\frac{1}{2}(a^{+}+a^{-})|k_{i:I}^{+},k_{i:I}^{-}\right)\\ p(b|k_{i:I}^{+},k_{i:I}^{-}) & =p\left(\frac{1}{2}(b^{+}+b^{-})|k_{i:I}^{+},k_{i:I}^{-}\right) \end{array}$$

**FIGURE 1:**
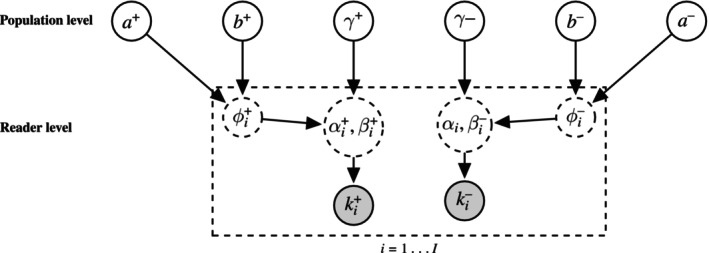
Schematic representation of the Bayesian hierarchical model architecture, illustrating the two‐level structure comprising the population and reader levels. The index i denotes individual readers among the I total. The parameters a (intercept), b (slope), and γ (overdispersion) are estimated independently for positive and negative cases using separate submodels. Dashed nodes correspond to latent variables, while solid nodes represent observed variables. Final parameter estimates for balanced accuracy are derived by averaging the posterior distributions from the positive and negative submodels.

A schematic representation of the hierarchical model structure using plate notation is provided in Figure 1.

In order to analyze weather posterior estimates vary significantly between the two imaging conditions, we compute different test statistics and compared there posterior distributions. For in case of an significant improvement in accuracy, the posterior effect size must be greater than zero. Thus the probability of an improvement in accuracy can be directly obtained from the posterior distribution of the effect size η as follows:

(13) $$p=\mathrm{Pr}(\eta >0|k_{i:I})$$

### 2.3 Bernoulli Model for Case‐Level Reading Data

Whereas we had previously modeled the aggregated counts of correctly classified cases, we propose a second model to accommodate case‐level reading data. For this model we assume that ratings are available for each individual case and reader, which allows us to take case specific variability as well as reader‐case interaction into account. By setting a fixed threshold for the ratings, we again dichotomize the rating to obtain a binary classification. The correct classification of case j by reader i and treatment condition t is then modeled as a Bernoulli random variable:

(14) $${\tilde{k}}_{ijt}∼\text{Bernoulli}(p_{ijt})$$

where $p_{ijt}$ is the probability of correctly classifying case j by reader i under treatment condition t. The probability of success $p_{ijt}$ is modeled using a logit link function as follows:

(15) $$p_{ijt}=\frac{1}{1+e^{-(a+b\cdot x_{t}+c_{j}+d_{ij})}}$$

where a is the intercept or baseline accuracy, b is the slope parameter representing the effect of the treatment condition, $c_{j}$ is a random effect for case j and $d_{ij}$ is a random effect for the interaction between reader i and case j. Similar to the previous model, balanced accuracy is obtained by conditioning the model on positive and negative cases separately and averaging the posterior distributions of the parameters across the two submodels. As we explicitly introduce case‐specific parameters to the model, the number of parameters increases consequently with the number of observations (i.e., number of cases). To prevent parameter estimation inconsistency, we apply hierarchical priors to the case‐specific parameters $c_{j}$ and $d_{ij}$, which allows for regularization of these parameters by pooling information across cases and readers.

### 2.4 Prior Distributions

The specification of prior distributions is a critical aspect of Bayesian modeling, as it encodes prior beliefs about the parameters before incorporating observed data. In this study, we considered three distinct prior settings:

1. informative priors,
2. weakly informative priors, and
3. diffuse (sometimes reffered to as non‐informative) priors.

Informative priors were selected based on existing literature and domain expertise, thereby constraining the parameter space to plausible values supported by prior evidence. The same prior distributions were applied to both the true positive and true negative models, as there is insufficient evidence from previous studies to justify different priors for these cases. The intercept and slope parameters were assigned normal distributions parameterized by their respective means μ and variances $\sigma^{2}$.

In case of the Beta‐Binomial model the overdispersion parameter γ was modeled assigned a truncated normal distribution to ensure it remained within the interval 0<γ<1.

For the Bernoulli model, the case‐specific parameters $c_{j}$ and reader‐case interaction parameters $d_{ij}$ were assigned only diffuse normal priors to allow for maximum flexibility in capturing case‐level variability and reader‐case interactions without imposing strong constraints on their values. This choice was motivated by the lack of prior information regarding the expected magnitude and variability of these parameters, as well as the desire to allow the data to inform their estimation without undue influence from the priors.

Prior predictive checks were conducted to evaluate the plausibility of the specified prior distributions for the intercept, slope, and overdispersion parameters. For each prior setting, six datasets were generated by sampling from the prior predictive distribution, simulating count outcomes for four readers without conditioning on observed data. For each reader, the median of 50 draws from the prior predictive distribution was computed and compared to the a priori expected accuracy.

### 2.5 Modelling and Posterior Distribution Evaluation

Bayesian modeling was implemented using the probabilistic programming framework PyMC version 5.22.0 [18]. Inference was performed using Markov Chain Monte Carlo (MCMC) sampling as implemented in PyMC, employing the No‐U‐Turn Sampler (NUTS). NUTS is an efficient variant of Hamiltonian Monte Carlo (HMC) that adaptively tunes the step size and trajectory length during sampling, thereby enhancing convergence and sampling efficiency, particularly in high‐dimensional parameter spaces. Posterior distributions were summarized by their central tendency and credible intervals, specifically reporting the posterior mode and the 95% Highest Density Interval (HDI). Model adequacy was assessed via posterior predictive checks (PPC) across all prior settings, wherein replicated datasets were generated from the posterior predictive distribution and compared to the observed data. The concordance between observed and simulated outcomes was evaluated by visualizing the 95% HDI of the posterior predictive distribution alongside the empirical counts of correctly classified cases for each reader and imaging condition.

Classical linear regression analyses were conducted using the statsmodels library version 0.14.4 [19].

For the real‐world dataset, the Bayesian modelling was complemented by the OR‐method for MRMC studies [13] to provide a point of comparison for the estimation of the two‐way partial ROC‐AUC. We used the OR method as implemented in the R package MRMCaov version 0.3.0 [20].

### 2.6 Evaluation of True Positive Rate, False Positive Rate, and Partial ROC‐AUC Analysis

One major advantage of proposed modeling approach is that accuracy is conditioned on positive and negative cases separately, which in turn allows for the analysis of the true positive rate (tpr) and true negative rate (tnr) independently. The estimated success probability can be directly interpreted as mean probability of correctly classifying a case. From the tnr one can derive the false positive rate (fpr) as fpr=1−tnr. The tnr and fpr can be used to construct a ROC curve by varying the decision threshold for the binary classification of the ratings. Similar to the analysis of the effect size in Equation (13), we can compute the probability of an improvement in tpr and tnr as follows:

(16) $$\begin{array}{ll} p_{tpr} & =\mathrm{Pr}(\Delta tpr>0|\overset{+}{\underset{i:I}{k}},k_{i:I}^{-}), \end{array}$$

(17) $$\begin{array}{ll} p_{fpr} & =\mathrm{Pr}(\Delta fpr>0|\overset{+}{\underset{i:I}{k}},k_{i:I}^{-}). \end{array}$$

with Δtpr and Δfpr denoting the posterior difference in tpr and fpr between the two imaging conditions, respectively.

However, because the prior on the population mean accuracy regularizes posterior estimates, extreme tpr and fpr values (i.e., near 0 and 1) are unlikely, and the resulting ROC curves are therefore confined to a restricted region of ROC space. Accordingly, comparison with classical ROC‐AUC approaches, such as the OR method, is only meaningful in terms of partial ROC‐AUC. For each comparison, we computed tpr and fpr values and identified the largest shared fpr interval. We then used the lower and upper bounds of this common interval to calculate partial ROC‐AUC for both the Bayesian model and the OR method. For the OR method, the figure of merit was the partial binormal AUC, which we compared with the trapezoidal partial AUC derived from Bayesian tpr and fpr estimates (Beta‐Binomial and Bernoulli models).

### 2.7 Simulation Study

Following the approach of Depaoli et al. [21], we conducted a simulation study by generating synthetic datasets for $k_{i}$ using fixed true values for the parameters a, b, and n for both true positive and true negative cases. The parameter values were selected to represent a realistic scenario, with $\Phi^{+}=0.75$, $\Phi^{-}=0.79$, $\eta^{+}=0.6$, and $\eta^{-}=0.4$, respectively. This results in a baseline balanced accuracy of Φ=0.77 and a balanced effect size of the enhancement technique of η=0.05. The number of trials per class was set to $n_{i}^{+}=80$ and $n_{i}^{-}=120$, mirroring the class imbalance commonly observed in MRMC studies and consistent with the real‐world dataset analyzed in this work.

To systematically investigate the influence of the number of readers and the degree of overdispersion on the deviation of posterior estimates from the true parameters, we considered I=[2,4,6,8,10,20,100,500,1000] and Γ=[0.1,0.2,0.5], corresponding to low, moderate and high overdispersion, respectively.

For each combination of I and Γ, 50 datasets were simulated, with $k_{i}^{+}$ and $k_{i}^{-}$ sampled from the Beta‐Binomial distribution parameterized by the true values. The Bayesian model was then fitted to each simulated dataset under all three prior settings to obtain parameter estimates. For comparison, a classical linear regression model was also fitted to the simulated data, specified as

(18) $$y_{i}=\Phi_{i}^{⋆}+\eta_{i}^{⋆}x_{i}+\epsilon_{i},\;i=1,\ldots ,I$$

where $y_{i}$ denotes the accuracy for reader i, $\Phi_{i}^{⋆}$ is the baseline accuracy, $\eta_{i}^{⋆}$ represents the effect size of the enhancement technique, $x_{i}$ is a binary indicator for the application of the enhancement, and $\epsilon_{i}$ is the error term. As with the Bayesian model, balanced accuracy was computed by averaging the parameter estimates for positive and negative cases. Note that the proposed classical linear regression model is a simplification without any explicit analysis of variance for case level effects or specification of reader‐case interactions, which reflects the structure of the Bayesian model for aggregated counts. Nevertheless it serves as a useful benchmark for evaluating the estimation performance and regularization characteristics of the Bayesian model. The direct comparison with a more sophisticated classical model was conducted in the real world example.

To quantitatively assess the estimation performance of the Bayesian and classical linear models, we calculated the mean absolute error (MAE):

(19) $$\text{MAE}=\frac{1}{N}\overset{N}{\underset{i=1}{\sum }}\left|\alpha_{i}-{\hat{\alpha }}_{i}\right|,$$

where $\alpha_{i}$ denotes the true parameter value, ${\hat{\alpha }}_{i}$ represents the corresponding model estimate, and N is the total number of simulated replicates. The MAE provides a summary measure of the average absolute deviation between the estimated and true parameter values across all simulations. We limited the simulation study to cover only the Beta‐Binomial model, since the focus of the study is on the estimation of population‐level parameters under the assumption of overdispersion, which is not explicitly modeled in the Bernoulli case.

### 2.8 Real‐World MRMC Study

The real‐world dataset analyzed in this study originates from a MRMC investigation assessing the diagnostic performance of pulmonary infiltrates in chest CXR imaging, both with and without the application of BSI enhancement. The primary objective of the original MRMC study was to evaluate whether a commercially available AI‐based BSI enhancement could improve the ability of junior radiologists to detect lung infiltrates indicative of COVID‐19 pneumonia. To this end, frontal CXR images from patients with confirmed COVID‐19 pneumonia were retrospectively retrieved from the Picture Archiving and Communication System of the emergency department at University Hospital Leipzig. The study cohort comprised 83 patients (mean age 58 years) with radiologically confirmed lung infiltrates and laboratory‐confirmed COVID‐19 pneumonia. An additional 129 age‐matched patients (mean age 58 years) without evidence of pulmonary infiltrates served as the control group, resulting in a total sample size of n=212 and a moderate class imbalance of approximately 2:3 (positive to negative cases).

Four junior radiologists independently assessed the likelihood of lung infiltrates consistent with COVID‐19 pneumonia on a continuous scale from 0 to 100, based on CXR images presented both with and without BSI enhancement. The classification threshold was set at a likelihood of 0.5 and explicitly communicated to all readers. The evaluation was conducted in two sequential stages: in the first stage, readers reviewed only the original CXR images; in the second stage, the original CXR was displayed alongside the corresponding BSI‐enhanced image for each patient. The order of case presentation was randomized. Readers were blinded to all clinical information and reference diagnoses and completed both stages in a single session without a washout period between conditions. For each reader and imaging condition, the numbers of correctly identified positive and negative cases were recorded and subsequently used as input for the Bayesian hierarchical model.

Ethical approval for this retrospective study was granted by the Review Board (number 20‐719). Due to the study's retrospective nature, written and informed consent was waved by the ethics board.

## 3 Results

### 3.1 Bayesian Analysis Computation

All sampling chains exhibited satisfactory convergence, with no divergences reported in the inference diagnostics. The GelmanRubin statistic ($\hat{R}$) was equal to 1 for all parameters and the effective sample size (ESS) in the bulk exceeded 10 000 for each parameter. Model calibration was assessed via simulation‐based calibration (SBC) following the approach of Talts et al. [22], using visual inspection of the empirical cumulative distribution function (ECDF) rank histograms. The ECDF histograms were approximately uniform for all parameters, indicating adequate coverage of the credible intervals under the specified model. Visualizations of the SBC diagnostics are provided in the Appendix A.

### 3.2 Prior Predictive Checks

**FIGURE 2:**
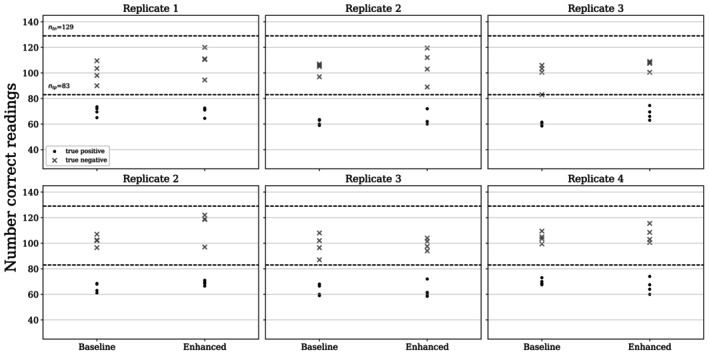
Prior predictive checks under the informative prior setting. Each panel displays six replicates of simulated counts for both baseline and enhanced imaging conditions, illustrating the range and central tendency of plausible outcomes generated by the prior distributions. The dashed lines indicate the number of true positiv (tp) and true negative (tn) cases included in the study.

Prior predictive checks were conducted for all three prior settings: diffuse, weakly informative and informative. The prior predictive simulations yielded a broad spectrum of plausible count outcomes, consistent with domain knowledge and expectations. For both imaging conditions (Baseline and Enhanced), the median ranges of simulated counts, when converted to accuracies, were centered around the a priori expected values, with occasional higher values attributable to the overdispersion specified in the priors. Figure 2 illustrates the six prior predictive replicates for the informative prior setting. Corresponding visualizations for the weakly informative and diffuse priors are provided in the Appendix B. These results indicate that the selected priors permit adequate variability while avoiding implausible extremes, thereby supporting their appropriateness for subsequent analyses.

### 3.3 Simulation Results

**FIGURE 3:**
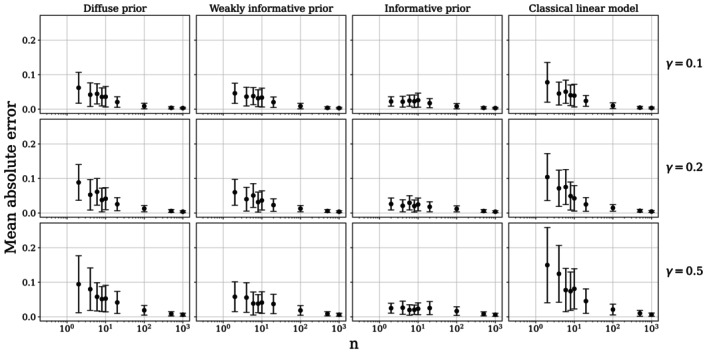
Mean absolute error (MAE) of the slope parameter estimates plotted over the number of readers for different amounts of overdispersion, comparing the Bayesian hierarchical model under different prior settings and the classical linear regression model. Results are averaged over 50 simulation replicates per condition. The Bayesian model demonstrates increased robustness and lower MAE, particularly in scenarios with small sample sizes and high overdispersion, when informative priors are used.

The primary objective of the simulation study was to assess the ability of the Bayesian hierarchical Beta‐Binomial model to recover the true population value of the slope parameter, representing the effect of imaging enhancement on balanced accuracy, under varying sample sizes and degrees of overdispersion. Figure 3 presents the MAE of the slope estimates plotted over the number of readers for different amounts of overdispersion and under different prior settings as well as for the classical linear regression model.

As expected, the MAE for all models decreases and converges toward zero with increasing sample size, indicating improved recovery of the true parameter value as more data become available. This convergence is more rapid for the classical linear model, whereas the Bayesian model exhibits greater regularization, particularly at small sample sizes (n≤10), due to the influence of the prior distributions. Informative priors yield the lowest MAE, reflecting the benefit of incorporating prior knowledge that is well‐aligned with the true parameter value. The classical linear model demonstrates the highest variability in MAE, attributable to the absence of regularization and increased susceptibility to extreme values, especially in the presence of high overdispersion.

With increasing overdispersion (γ), the advantage of the Bayesian approach becomes more pronounced: for moderate (γ=0.2) and high (γ=0.5) overdispersion, the Bayesian model achieves lower MAE and reduced variability compared to the classical linear model, particularly when informative priors are employed. Similar trends are observed for the intercept parameter. For the overdispersion parameter itself, differences in MAE between prior settings are less pronounced, but the MAE still decreases with increasing sample size and increases with higher overdispersion. Under informative priors, the MAE for the overdispersion parameter remains below 0.056±0.042 for low and moderate overdispersion, even at very small sample sizes (n<10). The highest MAE (0.093±0.06) is observed at n=2 under high overdispersion and informative priors.

### 3.4 Posterior Predictive Checks on Real‐World Data

**FIGURE 4:**
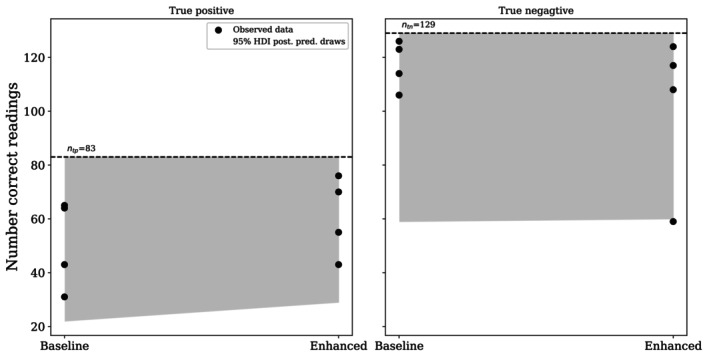
Posterior predictive checks for the real‐world MRMC dataset under informative priors. The figure displays the observed and model‐predicted numbers of correctly classified positive and negative cases for each reader and imaging condition (Baseline and Enhanced). Shaded regions represent the 95% highest density intervals (HDIs) of the posterior predictive distributions. The close correspondence between observed data and simulated intervals indicates good model calibration, with the exception of a single outlier in the Enhanced condition. The dashed lines indicate the number of true positiv (tp) and true negative (tn) cases included in the study.

PPC were performed under informative priors to assess the model's ability to reproduce the observed subject‐specific counts of correctly identified positive and negative cases for both imaging conditions (Baseline and Enhanced). As shown in Figure 4, the 95% HDIs of the simulated counts generally encompassed the empirical observations, indicating satisfactory model fit and calibration. Under diffuse and weakly informative priors, the posterior predictive intervals were broader, particularly for negative cases and thus included this extreme value. PPC plots for the weakly informative and diffuse prior settings are presented in the Appendix C. Across all prior settings, the model produced plausible ranges for the number of correctly classified cases, supporting the adequacy of the hierarchical specification.

To assess the sensitivity of the parameter estimates to prior specification, inference was performed under all three prior settings. Table 1 presents the posterior estimates for the negative, positive and balanced cases. The primary focus of the analysis is on the baseline accuracy Φ and the effect size η, as these are the key parameters of interest in the MRMC context described above. The baseline intercept estimate exhibits minimal variation across prior settings, reflecting the similarity of the prior distributions for a (see Table 2). The mean balanced accuracy at baseline is approximately 0.73, with class‐specific estimates of $\Phi^{-}\approx 0.80$ for negative cases and $\Phi^{+}\approx 0.65$ for positive cases. Under informative priors, the effect size η for balanced accuracy is estimated at approximately 0.03, with class‐specific effect sizes of $\eta^{-}\approx -0.01$ for negative cases and $\eta^{+}\approx 0.07$ for positive cases. The posterior probability that the effect size exceeds zero is p=0.70, suggesting a possible positive effect of the imaging enhancement technique on balanced accuracy. However, the sensitivity of the effect size estimate to the choice of prior is notable: under diffuse and weakly informative priors, the mean effect size is close to zero indicating no effect is under the model. Compared to the informative prior, the weakly informative and diffuse priors yield larger effect size estimates in both positive and negative directions, which balance out when averaged across classes. Specifically, the magnitude of the positive effect in positive cases is nearly offset by the negative effect in negative cases. As a result, the posterior probabilities that the effect size exceeds zero are p=0.52 and p=0.53 for the weakly informative and diffuse priors, respectively, indicating no substantial evidence for or against an effect of the imaging enhancement on balanced accuracy. The overdispersion parameter Γ ranges from 0.177 to 0.187 across prior settings, indicating a moderate degree of overdispersion in the data.

**TABLE 1:** Specification of prior distributions for the Bayesian model parameters under different prior settings. The N(μ,σ) notation denotes a normal distribution with mean μ and standard deviation σ. $N^{†}(0,\sigma )$ denotes a truncated normal distribution (with lowerbound 0, i.e., f(x)=0∀x<0) with zero mean and standard deviation σ. Priors for parameters a and b are applied to both the Beta‐Binomial and Bernoulli models. Case variation and reader‐case interaction priors where chosen to be diffuse, across all settings.

Model	Parameter	Diffuse	Weakly informative	Informative
—	a	𝒩(log(1),2)	$𝒩\left(\log\left(\frac{75}{25}\right),1\right)$	$𝒩\left(\log\left(\frac{75}{25}\right),1\right)$
	b	𝒩(0,2)	𝒩(0,1)	𝒩(0.2,0.5)
*Beta‐Binomial*	γ	${𝒩}^{†}(0,10^{2})$	${𝒩}^{†}(0,1)$	${𝒩}^{†}(0,0.5)$
*Bernoulli*	$c_{j}$	𝒩(0,2)	𝒩(0,2)	𝒩(0,2)
	$d_{ij}$	𝒩(0,2)	𝒩(0,2)	𝒩(0,2)

**TABLE 2:** Posterior estimates of the Bayesian hierarchical model parameters for the real‐world MRMC dataset under three different prior settings (informative, weakly informative, and diffuse). For each parameter, the table reports the posterior mode and the 95% highest density interval (HDI) in parentheses. Estimates are shown for balanced accuracy as well as for the true positive and true negative cases. The key parameters of interest are the baseline accuracy (ϕ), the effect size of imaging enhancement (η), and the overdispersion parameter (γ).

Estimate		Informative	Weakly informative	Diffuse
*balanced*				
	a	1.139 (0.692, 1.603)	1.138 (0.665, 1.606)	1.115 (0.622, 1.631)
	b	0.150 (−0.389, 0.669)	0.027 (−0.710, 0.772)	0.021 (−0.877, 0.878)
	Γ	0.177 (0.082, 0.281)	0.182 (0.084, 0.292)	0.187 (0.082, 0.299)
	Φ	0.735 (0.622, 0.845)	0.734 (0.617, 0.843)	0.729 (0.603, 0.845)
	η	0.027 (−0.075, 0.128)	0.005 (−0.138, 0.148)	0.005 (−0.171, 0.170)
*true positive*				
	$a^{+}$	0.740 (0.195, 1.331)	0.745 (0.158, 1.319)	0.687 (0.075, 1.316)
	$b^{+}$	0.348 (−0.353, 1.104)	0.402 (−0.586, 1.457)	0.496 (−0.712, 1.628)
	$\Gamma^{+}$	0.163 (0.052, 0.295)	0.170 (0.056, 0.309)	0.177 (0.055, 0.326)
	$\Phi^{+}$	0.651 (0.500, 0.798)	0.651 (0.492, 0.796)	0.638 (0.471, 0.798)
	$\eta^{+}$	0.074 (−0.089, 0.233)	0.085 (−0.138, 0.311)	0.106 (−0.154, 0.367)
*true negative*				
	$a^{-}$	1.539 (0.842, 2.299)	1.531 (0.777, 2.269)	1.544 (0.765, 2.366)
	$b^{-}$	−0.049 (−0.816, 0.706)	−0.347 (−1.450, 0.739)	−0.454 (−1.689, 0.868)
	$\Gamma^{-}$	0.190 (0.063, 0.353)	0.194 (0.054, 0.359)	0.196 (0.056, 0.372)
	$\Phi^{-}$	0.800 (0.666, 0.917)	0.798 (0.662, 0.921)	0.799 (0.646, 0.923)
	$\eta^{-}$	−0.007 (−0.127, 0.118)	−0.050 (−0.222, 0.127)	−0.064 (−0.261, 0.145)

*Note:* These proportions indicate the degree of uncertainty in the binary classifications and the sensitivity of the results to threshold choice: 14.8% of baseline ratings and 15.1% of enhanced ratings fell between 40 and 60.

### 3.5 Evaluation of True Positive and False Positive Rates

**FIGURE 5:**
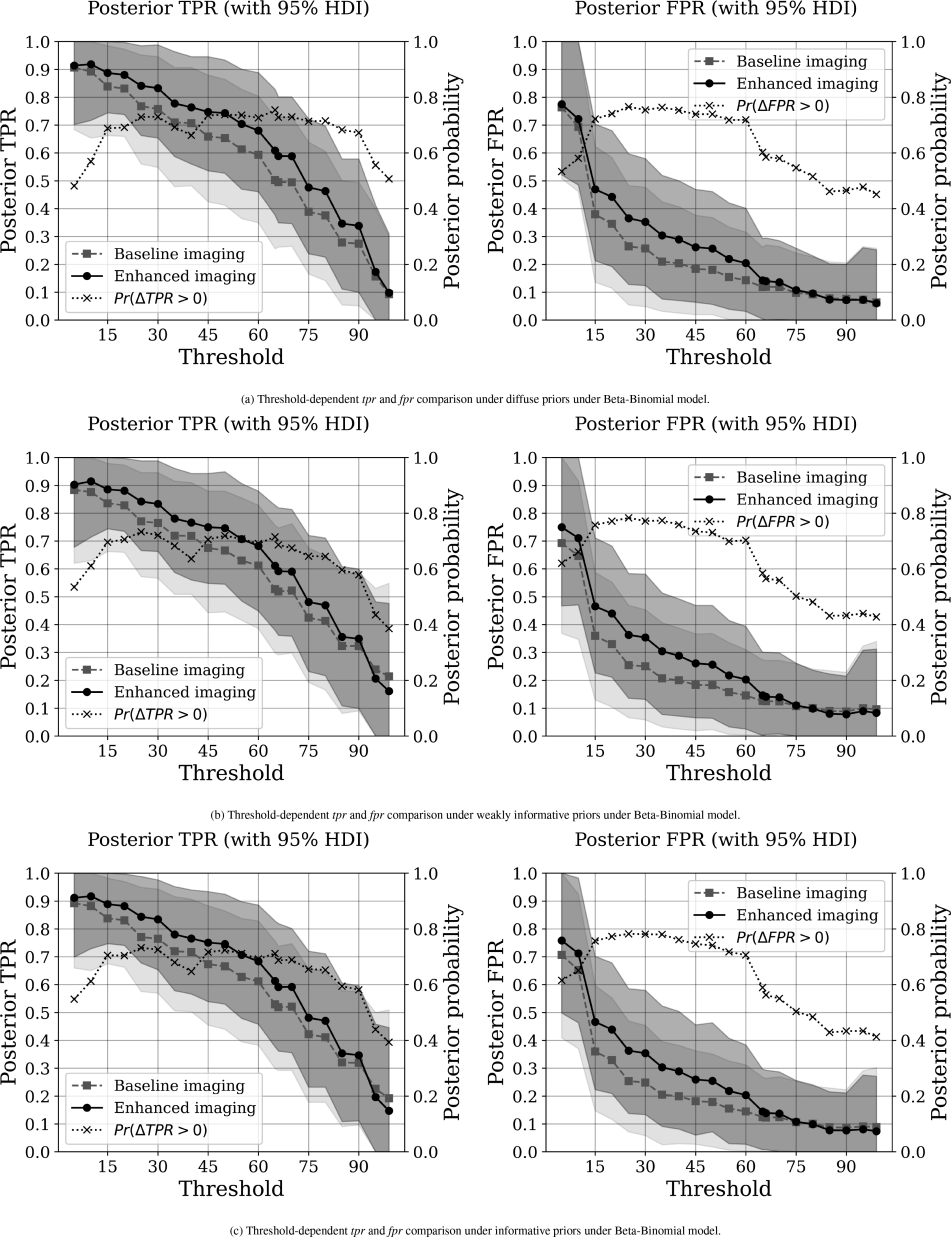
Comparison of threshold‐dependent posterior estimates of the true positive and false positive rates (left axis) across prior settings for the Beta‐Binomial model (top to bottom: diffuse, weakly informative, and informative priors). The right axis shows the corresponding posterior probabilities that tpr and fpr, respectively, are higher for enhanced imaging than for baseline imaging. (a) Threshold‐dependent tpr and fpr comparison under diffuse priors under Beta‐Binomial model. (b) Threshold‐dependent tpr and fpr comparison under weakly informative priors under Beta‐Binomial model. (c) Threshold‐dependent tpr and fpr comparison under informative priors under Beta‐Binomial model.

As can be seen in Figure 5 the posterior mean estimates obtained with the Beta‐Binomial model were largely stable across prior specifications. In contrast, uncertainty differed substantially by prior setting: diffuse and weakly informative priors produced noticeably wider HDI than the informative prior, consistent with the stronger regularization induced by informative priors.

For the threshold‐dependent comparison between enhanced and baseline imaging condition, the probability $\mathrm{Pr}(tpr_{baseline}>tpr_{enhanced})$ exceeded 0.7 only within an intermediate threshold range of approximately 15 to 70. Within this interval, a local reduction was observed between thresholds 30 and 45, where the probability decreased to about 0.65. A similar but narrower pattern was found for false positive rates: $\mathrm{Pr}(fpr_{enhanced}>fpr_{baseline})$ exceeded 0.7 primarily between thresholds 15 and 60, followed by a marked decline to values below 0.6 outside this region.

**FIGURE 6:**
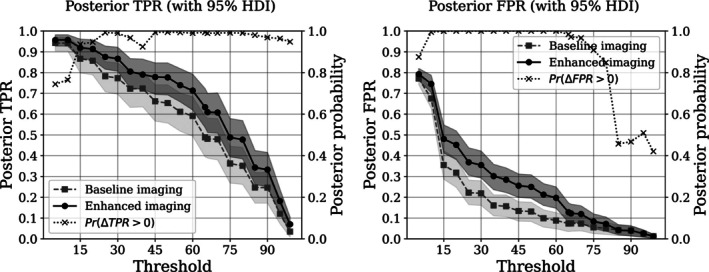
Comparison of threshold‐dependent posterior estimates of the true positive and false positive rates (left axis) for the Bernoulli model. The right axis shows the corresponding posterior probabilities that tpr and fpr, respectively, are higher for enhanced imaging than for baseline imaging. Since estimates are stable across prior settings, only the results for the diffuse prior setting are shown. Results for the remaining prior settings are provided in the Appendix.

Figure 6 shows, that under the Bernoulli model, the separation between baseline and enhanced conditions was more pronounced in the same threshold interval, with probabilities typically above 0.9 for both $\mathrm{Pr}(tpr_{enhanced}>tpr_{baseline})$ and $\mathrm{Pr}(fpr_{enhanced}>fpr_{baseline})$. Despite this stronger probabilistic separation, the absolute magnitude of the estimated differences remained modest, with maximum differences generally not exceeding 0.1. The Bernoulli model did not show clear variation across prior specifications. Consequently results where only depicted for one particular (diffuse) prior setting. Results from the remaining prior settings are provided in the Appendix D, which show similar patterns.

**FIGURE 7:**
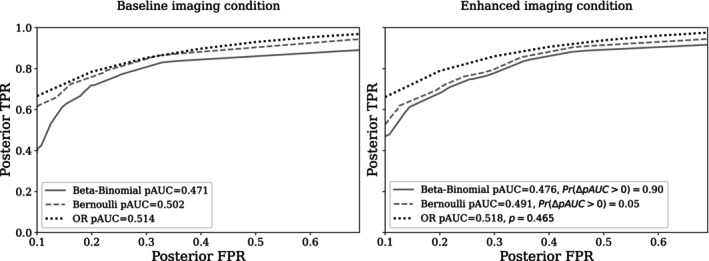
Comparison of partial ROC‐AUC estimates between the Bayesian hierarchical model (under informative prior settings) and the Obuchowski–Rockette method for the real‐world MRMC dataset. The figure displays the partial ROC curves and corresponding AUC values for both baseline and enhanced imaging conditions.

Across all prior settings, the partial ROC curves obtained from the Bayesian Beta‐Binomial model were consistently below those from the Obuchowski–Rockette (OR) method. Posterior ROC‐curves did not show substantial variation across prior settings, with the curves for the informative, weakly informative and diffuse priors largely overlapping. Consequently, only the results for the informative prior setting are depicted in Figure 7, with the remaining curves provided in the Appendix E. For baseline imaging, partial AUC estimates were 0.471 (informative prior) and 0.468 (weakly informative and diffuse priors), compared with 0.514 under the OR approach. For enhanced imaging, the corresponding partial AUC estimates were 0.475 (informative, weakly informative, and diffuse priors) versus 0.518 for OR. Consequently, the estimated improvement in partial AUC under the Beta‐Binomial model was small, ranging from approximately 0.004 to 0.005.

Although the posterior probability that the enhanced condition exceeded baseline was high in the Beta‐Binomial model (0.9 under weakly informative and informative priors, and 1.0 under diffuse priors), the uncertainty intervals for the partial AUC difference included zero across all prior settings. In line with this, the corresponding frequentist comparison did not indicate statistical significance (p=0.465).

For the Bernoulli model, the partial ROC curves were closer to the OR results, particularly in the baseline condition. However, the direction of the contrast differed from the Beta‐Binomial model and OR results: partial AUC was slightly lower for enhanced imaging (0.491) than for baseline imaging (0.502), with a posterior probability of only 0.05 that enhanced imaging outperformed baseline. As with the Beta‐Binomial model, the HDIs for the between‐condition partial AUC difference included zero, indicating no statistically significant difference in diagnostic performance between baseline and enhanced imaging based on partial ROC‐AUC.

## 4 Discussion

The simulation study demonstrated that the Bayesian hierarchical model reliably recovers true population parameters, even under conditions of limited sample size and substantial overdispersion. Compared to classical modeling, our approach exhibits greater robustness to outliers and extreme values, particularly when informative priors are employed. The integration of prior knowledge enables regularization of parameter estimates, resulting in lower MAE relative to the true values, especially in scenarios characterized by high overdispersion. Analysis of the real‐world MRMC dataset suggests a positive effect of the imaging enhancement technique on balanced accuracy, with a posterior probability of the effect size exceeding zero of p=0.70 under informative priors. Balanced accuracy, by accounting for class imbalance, provides a more reliable assessment of classification performance than conventional accuracy, as evidenced by the consistent results across positive and negative cases.

In comparison to prior studies investigating the impact of CAD and BSI on reader performance, the observed effect size aligns with previously reported small but positive improvements in diagnostic accuracy [23, 24, 25, 26, 27, 28, 29, 30]. The partial ROC‐AUC differences observed in the present analysis did not show particular evidence for an considerable performance gain. In contrast, Freedman et al. reported an increase in localization ROC‐AUC from 0.46 to 0.56 with BSI‐enhanced CAD for lung nodule detection [31], a larger effect than considered in the present simulation study. However, their analysis did not account for class imbalance, which may have biased the reported metric. The present model explicitly addresses this limitation by employing balanced accuracy.

Overdispersion, as quantified by the parameter γ, is infrequently addressed in the MRMC literature. Only a limited number of studies have considered inter‐reader variability, often using κ statistics to assess agreement [32]. The moderate overdispersion observed in this study is consistent with previously reported weak to moderate inter‐reader agreement. As demonstrated in the simulation study, even moderate overdispersion (γ=0.2) can introduce bias in parameter estimates if not appropriately modeled. Thus, accounting for overdispersion is essential for valid inference in MRMC studies.

A key distinction emerged between the Beta‐Binomial and Bernoulli model formulations, particularly in the magnitude of uncertainty quantified in the threshold‐dependent TPR and FPR analyses. The narrower HDI observed in the Bernoulli model reflect its fundamentally different data aggregation strategy. The Beta‐Binomial model aggregates data aggressively, grouping observations only by reader and treatment condition, yielding merely 8 observations per condition. In contrast, the Bernoulli model retains the full case‐level panel structure, resulting in 1032 observations for the negative cases and 664 for the positive cases. This substantially increased information density enables more precise posterior estimates of reader‐case‐specific performance. Unexplained heterogeneity in the Beta‐Binomial model is absorbed into the overdispersion parameter γ, whereas the Bernoulli model explicitly models case‐level and reader‐case interaction effects. Consequently, the tpr and fpr posterior distributions reflect pooling across a far denser observation structure in the Bernoulli specification. The broader posterior uncertainty produced by the Beta‐Binomial model can nevertheless beconsidered a feature rather than a limitation: by absorbing unmodeled heterogeneity into a single overdispersion parameter, the model avoids overconfident conclusions in settings where the precise structure of reader‐case interactions and sources of case variability are not fully characterized.

A potential concern with the Bernoulli model is that the number of parameters grows with the number of cases, which might lead to inconsistent estimation under frequentist maximum likelihood approaches. However, the hierarchical Bayesian framework mitigates this issue by regularizing the parameter explosion through a lower‐dimensional prior structure. Random effects for cases and reader‐case interactions are modeled as draws from shared normal distributions, with effective complexity controlled by the hyperparameter estimates. This regularization ensures stable and interpretable inference despite the larger parameter space.

An important finding of this study is the apparent discrepancy between global and local performance summaries. While the partial ROC‐AUC analysis did not indicate a clear overall performance gain with image enhancement, the threshold‐dependent analysis revealed clinically relevant differences in specific operating regions. In particular, both the tpr and fpr were up to approximately 10 percentage points higher in selected threshold intervals under the enhanced condition. This pattern suggests that enhancement may not uniformly improve discrimination across the full decision range, but can meaningfully shift performance at specific working points.

These results have direct implications for clinical deployment. Threshold selection is not a purely technical choice; it must be aligned with the clinical context and the relative consequences of false negatives versus false positives. For example, in scenarios where missing positive cases is particularly harmful, thresholds can be chosen to prioritize sensitivity, whereas settings with high downstream costs of over‐calling may favor higher specificity. The threshold‐dependent posterior estimates provided by our framework support this decision process by explicitly quantifying expected gains and trade‐offs at candidate operating points.

A key strength of the proposed Bayesian approach is that it enables this operating‐point‐specific evaluation together with uncertainty quantification. Rather than relying solely on a single aggregate metric, the model provides probabilistic evidence for differences in TPR and FPR across thresholds, allowing decision‐makers to identify regions where improvement is most plausible and where uncertainty remains substantial. This supports more transparent and context‐aware interpretation of enhancement effects in MRMC studies.

Despite the methodological advantages, the effect size observed in the real‐world example is small and the evidence supporting a positive effect remains limited due to the modest sample size. Consequently, parameter estimates particularly the effect size should be interpreted with caution, as they are sensitive to prior specification.

## 5 Conclusion

In this study, we introduced a Bayesian hierarchical model for estimating balanced accuracy in binary classification, with a focus on MRMC studies characterized by small sample sizes and high overdispersion. The model incorporates prior knowledge through flexible prior settings, enabling regularization and rigorous quantification of uncertainty. Simulation results demonstrated the model's robustness in recovering true population parameters under challenging conditions. Application to a real‐world MRMC study of pulmonary infiltrate detection in CXR imaging with and without BSI enhancement illustrated the model's practical utility. The model flexibility allowed for a nuanced analysis of diagnostic performance, including threshold‐dependent TPR and FPR comparisons which revealed relevant differences in specific operating regions despite a lack of significant improvement in global performance metrics such as partial ROC‐AUC. However, the observed overdispersion and limited sample size underscore the need for larger studies to draw definitive conclusions about possible diagnostic improvements induced by BSI enhancement. Our findings highlight the importance of accounting for overdispersion and sample size in MRMC study design and demonstrate that, when the number of readers is limited, the use of well‐justified priors, based on expert knowledge or prior studies, can yield more robust parameter estimates.

## Author Contributions

Conceptualization was performed by Paul‐Philipp Jacobs, Ingo G. Steffen, Constantin Ehrengut, and Timm Denecke. Methodology was developed by Paul‐Philipp Jacobs and Ingo G. Steffen. Formal analysis was carried out by Paul‐Philipp Jacobs. Investigation was conducted by Constantin Ehrengut, Susann Bräuer, and Hans‐Jonas Meyer. Resources were provided by Paul‐Philipp Jacobs, Timm Denecke, and Hans‐Jonas Meyer. The original draft was written by Paul‐Philipp Jacobs and Ingo G. Steffen. Writing, review, and editing were performed by Paul‐Philipp Jacobs, Ingo G. Steffen, Constantin Ehrengut, Hans‐Jonas Meyer, and Timm Denecke. Visualization was prepared by Paul‐Philipp Jacobs and Ingo G. Steffen. Supervision was provided by Hans‐Jonas Meyer and Timm Denecke. Project administration was handled by Constantin Ehrengut and Timm Denecke. Funding acquisition was secured by Timm Denecke.

## Funding

This work was supported by the Bundesministerium für Bildung und Forschung (Grant No. 01KX2121).

## Disclosure

The authors have nothing to report.

## Conflicts of Interest

The authors declare no conflicts of interest.

## Figures and Tables

**FIGURE A1:**
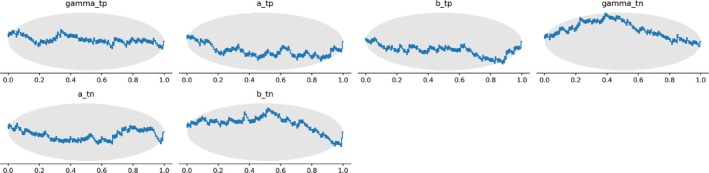
Simulation Based Calibration (SBC) results for the model. The plot shows the empirical cumulative distribution function (ECDF) of the simulated p‐values from the SBC procedure. A uniform distribution across the range [0, 1] indicates good calibration.

**FIGURE B1:**
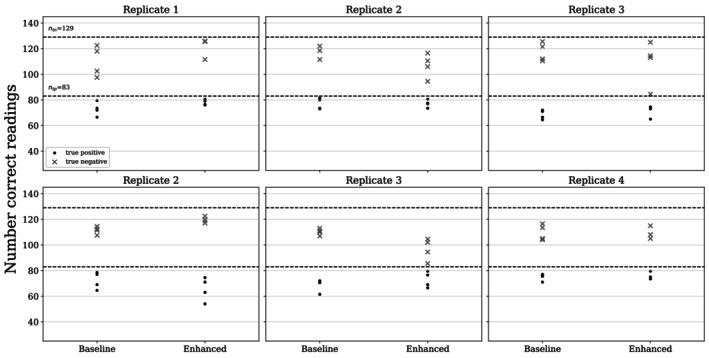
Prior predictive check for the model with **weakly informative priors**. Observed data are compared to simulated data from the prior predictive distribution to assess model fit and the influence of the priors. The dashed lines indicate the number of true positiv (tp) and true negative (tn) cases included in the study.

**FIGURE B2:**
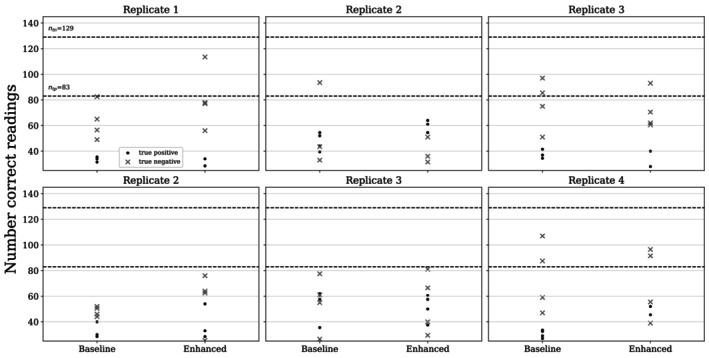
Prior predictive check for the model with **diffuse priors**. Observed data are compared to simulated data from the prior predictive distribution to assess model fit and the influence of the priors. The dashed lines indicate the number of true positiv (tp) and true negative (tn) cases included in the study.

**FIGURE C1:**
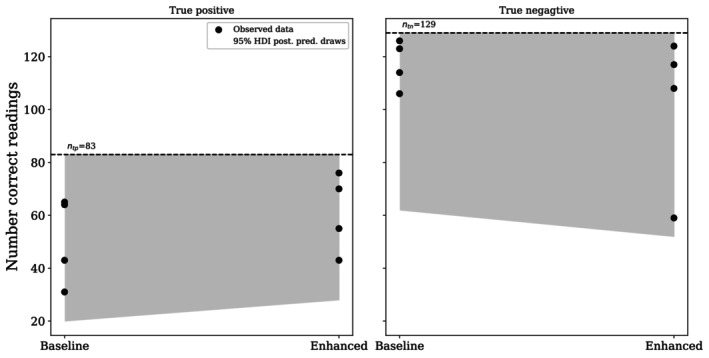
Posterior predictive check for the model under weakly informative priors. Real‐World observed data are compared to simulated data from the posterior predictive distribution to assess model fit and adequacy. The dashed lines indicate the number of true positiv (tp) and true negative (tn) cases included in the study.

**FIGURE C2:**
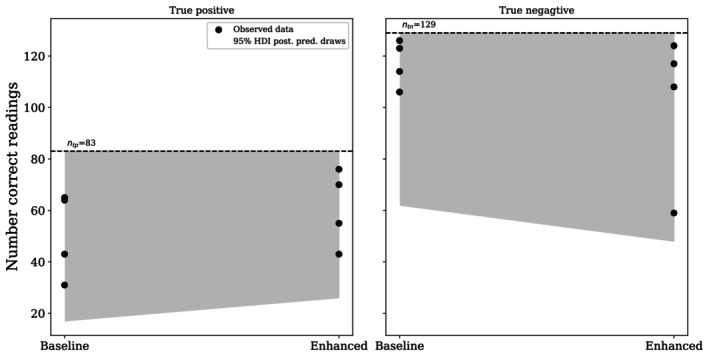
Posterior predictive check for the model under diffuse informative priors. Real‐world observed data are compared to simulated data from the posterior predictive distribution to assess model fit and adequacy. The dashed lines indicate the number of true positiv (tp) and true negative (tn) cases included in the study.

**FIGURE D1:**
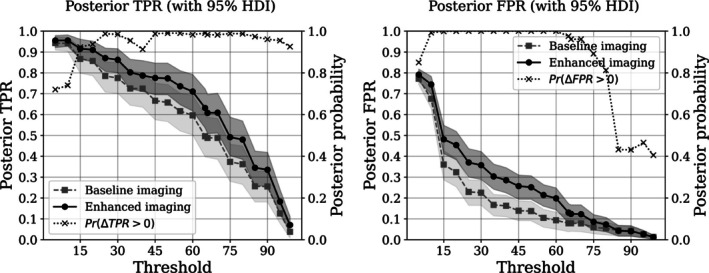
Posterior FPR, TPR for the Bernoulli model using real‐world MRMC data with weakly informative priors.

**FIGURE D2:**
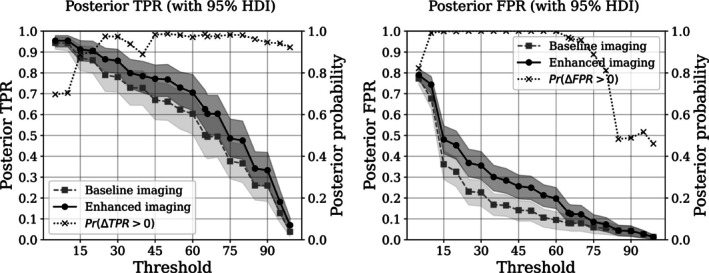
Posterior FPR, TPR for the Bernoulli model using real‐world MRMC data with informative priors.

**FIGURE E1:**
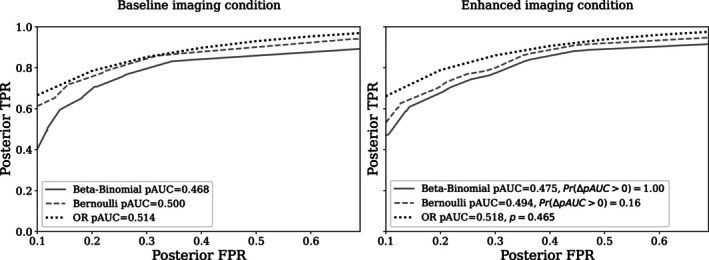
Posterior partial AUC using real‐world MRMC data with diffuse priors.

**FIGURE E2:**
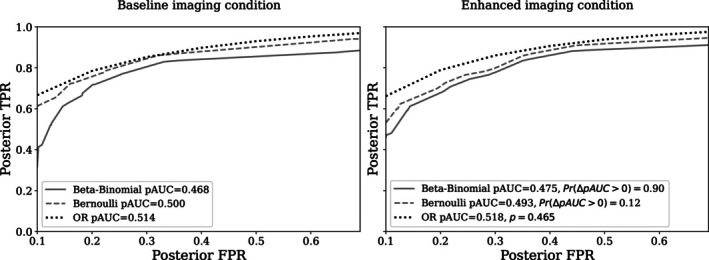
Posterior partial AUC using real‐world MRMC data with weakly informative priors.

## Data Availability

The data that support the findings of this study are openly available in BayBacMRMC at https://github.com/PeePeeJay/BayBacMRMC.

## Appendix A Visualization of the Simulation Based Calibration Results

See Figure A1.

## Appendix B Visualization of the Prior Predictive Check Results Under Diffuse and Weakly Informative Priors

See Figures B1 and B2.

## Appendix C Visualization of the Posterior Predictive Check Results Using Real‐World MRMC Data

See Figures C1 and C2.

## Appendix D Visualization of the Posterior FPR, TPR for the Bernoulli Model Using Real‐World MRMC Data With Informative and Weakly Informative Priors

See Figures D1 and D2.

## Appendix E Visualization of the Posterior Partial AUC Using Real‐World MRMC Data With Diffuseand Weakly Informative Priors

See Figures E1 and E2.

## References

[1] C. Wang , A. Elazab , J. Wu , and Q. Hu , “Lung Nodule Classification Using Deep Feature Fusion in Chest Radiography,” Computerized Medical Imaging and Graphics 57 (2017): 10–18, 10.1016/j.compmedimag.2016.11.004. 27986379

[2] W. Yang , Y. Chen , Y. Liu , et al., “Cascade of Multi‐Scale Convolutional Neural Networks for Bone Suppression of Chest Radiographs in Gradient Domain,” Medical Image Analysis 35 (2017): 421–433, 10.1016/j.media.2016.08.004. 27589577

[3] R. Manickavasagam , S. Selvan , and M. Selvan , “CAD System for Lung Nodule Detection Using Deep Learning With CNN,” Medical & Biological Engineering & Computing 60, no. 1 (2022): 221–228, 10.1007/s11517-021-02462-3. 34811644

[4] D. Ribli , A. Horváth , Z. Unger , P. Pollner , and I. Csabai , “Detecting and Classifying Lesions in Mammograms With Deep Learning,” Scientific Reports 8, no. 1 (2018): 4165, 10.1038/s41598-018-22437-z. 29545529 PMC5854668

[5] C. Pitarch , V. Ribas , and A. Vellido , “AI‐Based Glioma Grading for a Trustworthy Diagnosis: An Analytical Pipeline for Improved Reliability,” Cancers 15, no. 13 (2023): 3369, 10.3390/cancers15133369. 37444479 PMC10341156

[6] K. Matsubara , M. Ibaraki , M. Nemoto , H. Watabe , and Y. Kimura , “A Review on AI in PET Imaging,” Annals of Nuclear Medicine 36, no. 2 (2022): 133–143, 10.1007/s12149-021-01710-8. 35029818

[7] B. Huang , J. Sollee , Y. H. Luo , et al., “Prediction of Lung Malignancy Progression and Survival With Machine Learning Based on Pre‐Treatment FDG‐PET/CT,” eBioMedicine 82 (2022): 104127, 10.1016/j.ebiom.2022.104127. 35810561 PMC9278031

[8] Y. T. Shen , L. Chen , W. W. Yue , and H. X. Xu , “Artificial Intelligence in Ultrasound,” European Journal of Radiology 139 (2021): 109717, 10.1016/j.ejrad.2021.109717. 33962110

[9] N. A. Obuchowski and J. Bullen , “Multireader Diagnostic Accuracy Imaging Studies: Fundamentals of Design and Analysis,” Radiology 303, no. 1 (2022): 26–34, 10.1148/radiol.211593. 35166584

[10] T. Dendumrongsup , A. A. Plumb , S. Halligan , T. R. Fanshawe , D. G. Altman , and S. Mallett , “Multi‐Reader Multi‐Case Studies Using the Area Under the Receiver Operator Characteristic Curve as a Measure of Diagnostic Accuracy: Systematic Review With a Focus on Quality of Data Reporting,” PLoS One 9, no. 12 (2014): e116018, 10.1371/journal.pone.0116018. 25541977 PMC4277459

[11] D. Fletcher , P. W. Dillingham , and M. Parry , “A Simple and Robust Approach to Bayesian Modelling of Overdispersed Data,” Environmental and Ecological Statistics 30, no. 2 (2023): 289–308, 10.1007/s10651-023-00567-6.

[12] P. Hougaard , M. L. Lee , and G. A. Whitmore , “Analysis of Overdispersed Count Data by Mixtures of Poisson Variables and Poisson Processes,” Biometrics 53, no. 4 (1997): 1225–1238. 9423246

[13] N. A. Obuchowski , S. V. Beiden , K. S. Berbaum , et al., “Multireader, Multicase Receiver Operating Characteristic Analysis: An Empirical Comparison of Five Methods1,” Academic Radiology 11, no. 9 (2004): 980–995, 10.1016/j.acra.2004.04.014. 15350579

[14] J. K. Kruschke , “Bayesian Analysis Reporting Guidelines,” Nature Human Behaviour 5, no. 10 (2021): 1282–1291, 10.1038/s41562-021-01177-7. PMC8526359 34400814

[15] C. E. Metz , “Basic Principles of ROC Analysis,” Seminars in Nuclear Medicine 8, no. 4 (1978): 283–298, 10.1016/S0001-2998(78)80014-2. 112681

[16] K. H. Brodersen , C. S. Ong , K. E. Stephan , and J. M. Buhmann , “The Balanced Accuracy and Its Posterior Distribution,” in *2010 20th International Conference on Pattern Recognition*. (IEEE, 01–02 August, 2010).

[17] K. H. Brodersen , C. Mathys , J. R. Chumbley , et al., “Bayesian Mixed‐Effects Inference on Classification Performance in Hierarchical Data Sets,” Journal of Machine Learning Research 13, no. 101 (2012): 3133–3176.

[18] O. Abril‐Pla , V. Andreani , C. Carroll , et al., “PyMC: A Modern, and Comprehensive Probabilistic Programming Framework in Python,” PeerJ Computer Science 9 (2023): e1516, 10.7717/peerj-cs.1516. PMC10495961 37705656

[19] S. Seabold and J. Perktold , “Statsmodels: Econometric and Statistical Modeling with Python,” *scipy*, (2010), 10.25080/Majora-92bf1922-011.

[20] B. J. Smith and S. L. Hillis , “Multi‐Reader Multi‐Case Analysis of Variance Software for Diagnostic Performance Comparison of Imaging Modalities,” in F. Samuelson and S. Taylor‐Phillips *Proceedings of SPIE 11316, Medical Imaging 2020: Image Perception, Observer Performance, and Technology Assessment*, 113160K, (2020), 10.1117/12.2549075. PMC7190386 32351258

[21] S. Depaoli , S. D. Winter , and M. Visser , “The Importance of Prior Sensitivity Analysis in Bayesian Statistics: Demonstrations Using an Interactive Shiny App,” Frontiers in Psychology 11 (2020): 11, 10.3389/fpsyg.2020.608045. 33324306 PMC7721677

[22] S. Talts , M. Betancourt , D. Simpson , A. Vehtari , and A. Gelman , “Validating Bayesian Inference Algorithms With Simulation‐Based Calibration,” arXiv:1804.06788, (2020) [stat], 10.48550/arXiv.1804.06788.

[23] M. D. Dorrius , d. M. C. J. Weide , O. vPMA , R. M. Pijnappel , and M. Oudkerk , “Computer‐Aided Detection in Breast MRI: A Systematic Review and Meta‐Analysis,” European Radiology 21, no. 8 (2011): 1600–1608, 10.1007/s00330-011-2091-9. 21404134 PMC3128262

[24] R. A. Benedikt , J. E. Boatsman , C. A. Swann , A. D. Kirkpatrick , and A. Y. Toledano , “Concurrent Computer‐Aided Detection Improves Reading Time of Digital Breast Tomosynthesis and Maintains Interpretation Performance in a Multireader Multicase Study,” American Journal of Roentgenology 210, no. 3 (2018): 685–694, 10.2214/AJR.17.18185. 29064756

[25] G. S. Hong , K. H. Do , and C. W. Lee , “Added Value of Bone Suppression Image in the Detection of Subtle Lung Lesions on Chest Radiographs With Regard to Reader's Expertise,” Journal of Korean Medical Science 34, no. 38 (2019): e250, 10.3346/jkms.2019.34.e250. 31583870 PMC6776835

[26] v. J. Berg , S. Young , H. Carolus , et al., “A Novel Bone Suppression Method That Improves Lung Nodule Detection,” International Journal of Computer Assisted Radiology and Surgery 11, no. 4 (2016): 641–655, 10.1007/s11548-015-1278-y. 26337439

[27] S. Schalekamp , B. Ginneken , L. Meiss , et al., “Bone Suppressed Images Improve Radiologists Detection Performance for Pulmonary Nodules in Chest Radiographs,” European Journal of Radiology 82, no. 12 (2013): 2399–2405, 10.1016/j.ejrad.2013.09.016. 24113431

[28] F. Li , R. Engelmann , L. Pesce , S. G. Armato , and H. MacMahon , “Improved Detection of Focal Pneumonia by Chest Radiography With Bone Suppression Imaging,” European Radiology 22, no. 12 (2012): 2729–2735, 10.1007/s00330-012-2550-y. 22763504

[29] F. Li , R. Engelmann , L. Pesce , K. Doi , C. E. Metz , and H. MacMahon , “Small Lung Cancers: Improved Detection by Use of Bone Suppression Imaging Comparison With Dual‐Energy Subtraction Chest Radiography,” Radiology 261, no. 3 (2011): 937–949, 10.1148/radiol.11110192. 21946054 PMC6940009

[30] S. Oda , K. Awai , K. Suzuki , et al., “Performance of Radiologists in Detection of Small Pulmonary Nodules on Chest Radiographs: Effect of Rib Suppression With a Massive‐Training Artificial Neural Network,” American Journal of Roentgenology 193, no. 5 (2009): W397–W402, 10.2214/AJR.09.2431. 19843717

[31] M. T. Freedman , L. SCB , J. C. Seibel , and C. M. Bromley , “Lung Nodules: Improved Detection With Software That Suppresses the Rib and Clavicle on Chest Radiographs,” Radiology 260, no. 1 (2011): 265–273, 10.1148/radiol.11100153. 21493789

[32] J. R. Landis and G. G. Koch , “An Application of Hierarchical Kappa‐Type Statistics in the Assessment of Majority Agreement Among Multiple Observers,” Biometrics 33, no. 2 (1977): 363–374, 10.2307/2529786. 884196

